# Delayed Orbital Floor Reconstruction Using Mirroring Technique and Patient-Specific Implants: Proof of Concept

**DOI:** 10.3390/jpm14050459

**Published:** 2024-04-26

**Authors:** Diana D’Alpaos, Giovanni Badiali, Francesco Ceccariglia, Achille Tarsitano

**Affiliations:** 1 Oral and Maxillo-Facial Surgery Unit, IRCCS Azienda Ospedaliero—University of Bologna, Via Albertoni 15, 40138 Bologna, Italy; giovanni.badiali@unibo.it (G.B.); francesc.ceccarigli2@unibo.it (F.C.); achille.tarsitano2@unibo.it (A.T.); 2 Department of Biomedical and Neuromotor Science, Alma Mater Studiorum—University of Bologna, 40138 Bologna, Italy

**Keywords:** orbital reconstruction, enophthalmos, CAD-CAM technique, titanium mesh, HDPE implant, personalized surgery

## Abstract

Enophthalmos is a severe complication of primary reconstruction following orbital floor fractures, oncological resections, or maxillo-facial syndromes. The goal of secondary orbital reconstruction is to regain a symmetrical globe position to restore function and aesthetics. In this article, we present a method of computer-assisted orbital floor reconstruction using a mirroring technique and a custom-made titanium or high-density polyethylene mesh printed using computer-aided manufacturing techniques. This reconstructive protocol involves four steps: mirroring of the healthy orbit computer tomography files at the contralateral affected site, virtual design of a customized implant, computer-assisted manufacturing (CAM) of the implant using Direct Metal Laser Sintering (DMLS) or Computer Numerical Control (CNC) methods, and surgical insertion of the device. Clinical outcomes were assessed using 3dMD photogrammetry and computed tomography measures in 13 treated patients and compared to a control group treated with stock implants. An improvement of 3.04 mm (range 0.3–6 mm) in globe protrusion was obtained for the patients treated with patient-specific implants (PSI), and no major complications have been registered. The technique described here appears to be a viable method for correcting complex orbital floor defects needing delayed reconstruction.

## 1. Introduction

Fractures of the orbit alone or in combination with other facial skeleton fractures are common in cases of midface trauma: the orbital floor is affected in up to 57% of midface fractures [1].

They can lead to entrapment of extraocular muscles and/or intermuscular septum and loss of orbital volume with different clinical symptoms and/or complications, such as diplopia, enophthalmos, ocular dystopia, subconjunctival bleeding, ocular contusion, etc., which can lead to functional and morphological deficits, resulting in social and occupational problems.

A complete restoration of orbital contents with an anatomically correct reconstruction of orbital walls is necessary to avoid functional deficits and for restoration of anatomical relations and morphology [2]. The main aims of post-traumatic orbital reconstruction include both the release and reduction of herniated or prolapsed soft tissue, accurate and stable reconstruction of the orbital walls, restoration of native orbital volume and preservation of vital structures such as muscles and nerves [2].

Orbital dystopia and enophthalmos are the most important sequelae of blow-out orbital floor fractures.

Significant enophthalmos after orbital injury and floor fracture may not be immediately visible because of edema and swelling of the surrounding orbital tissues. This swelling and hemorrhage could even cause proptosis on the affected side. A retrospective noncomparative study by Hawes and Dortzbach [3] reported that computed tomography (CT) scan is a useful tool for predicting postinjury enophthalmos. The authors recommended surgical repair within 2 weeks if greater than one-half of the orbital floor is depressed. They reported less satisfactory enophthalmos repair with later correction because of presumed fat atrophy and scarring of the orbital fat to the maxillary antrum, making late repair unsatisfactory [4].

However, medical literature highlights that immediate surgical treatment is needed in trapdoor fractures or in cases involving extraocular muscle function showing up as enophthalmos or hypoglobus. Otherwise, observation can be recommended to allow for the resolution of edema before a definitive evaluation of the need for treatment. If required, surgery is usually performed within 7–14 days of injury. Otherwise, it is recommended that surgery be delayed to allow the tissues to heal before further intervention [5].

Reconstruction of the orbital wall(s) is also required during or after midface oncologic surgery following radical surgery for tumors involving the orbit, and it can be considered uniquely complex. Secondary procedures can be challenging in correcting structural derangements, especially if adjuvant radiation is administered, and so care must be taken to select the optimal reconstructive option at the same operative time for demolition, whenever it is possible.

The main aim of delayed orbital reconstruction is to restore a symmetrical globe position to recover function and morphology.

Orbital floor reconstruction can be achieved using a variety of different methods and materials: a review made by Avashia et al. [6], including 3457 distinct orbital floor reconstructions, showed inconclusive data on any biomaterial/implant as absolute best fit for orbital floor reconstruction. As demonstrated, each material has its unique properties, and the choice of one over another is often related to the kind of defect other than the surgeon’s preference.

For example, the use of biological materials such as fervens bones, autogenous bones, or collagen membranes (Lyoplant^®^, BBraun SPA, Melsungen, Germany) offers the potential advantages of better biocompatibility but comes at the cost of donor-site morbidity.

Conversely, the use of synthetic implants, such as titanium meshes or high-density polyethylene foils or implants, has historically been associated with higher rates of complications, especially infections and bad tolerance of the tissue to the presence of a foreign non-organic material [7].

Non-biologic materials are indeed capable of providing strength and durability, are not prone to resorption, and do create a potential donor site morbidity; however, they are prone to infection as they never mucosalize when they abut sinuses [8] and can undergo superficilization and/or cutaneous extrusion over time.

The advantages of titanium mesh plates include availability, biocompatibility, ease of intraoperative contouring, and rigid fixation [9].

Unfortunately, these implants are not always easily positioned: the placement of the mesh deep within the orbital cone can be challenging, especially in secondary orbital reconstruction, when scarring sometimes obscures the identification of certain stable anatomical landmarks.

Custom-made titanium implants using computer-assisted designs have enabled surgeons to achieve optimal reconstruction in areas of limited visibility and protection of vital structures [10].

Computer-assisted design and computer-assisted manufacturing today are largely applied in maxillo-facial reconstructive surgery, thus increasing the accuracy and allowing better clinical outcomes to be achieved [11,12].

Consequently, since the early 2000s, there has been a growing use of individual implants in orbital floor reconstruction, clearly demonstrating them to be less time-consuming and more accurate if compared to “free hand” modeling of the implant [13].

With the increasing interest in Computer-Assisted Surgery (CAS), there has been a large application of patient-specific reconstruction of orbital floor defects, and a large number of research papers have demonstrated the advantages existing in the application of patient-specific implants (PSI) for orbital reconstructions [14,15].

CAS for orbital reconstruction can be achieved through manual preoperative molding of the implant based on a patient’s stereolithographic 3D model (produced on the basis of CT scans). This technology increases the orbital surgeon’s options in managing complex orbital pathology [16].

Another form of CAS in orbital reconstruction is Computer Aided Design–Computer Assisted Manufacturing (CAD-CAM) procedures, which come in the form of preoperative digital design of the implant based on the mirroring of the uninjured 3D reconstruction of the orbit on the contralateral affected side; the digital implant is then manufactured thanks to a computer-assisted technique, such as direct metal laser sintering or computer numerical control.

The aim of the present prospective clinical study, which follows the previous publication of a preliminary study realized in our center [17], is to assess the clinical outcomes after CAD-CAM delayed reconstruction of the floor of the orbit, thus more specifically addressing restoration of the globe position and resolution of enophthalmos.

## 2. Materials and Methods

This study was approved by local ethical board (approval no. 57/2011/O/Disp). and conducted on 13 patients who underwent surgical reconstruction of the orbital floor using computer-assisted design-computer-assisted manufacturing (CAD-CAM) method. Only secondary orbital floor reconstructions were enrolled in this study (≥8 months between primary event, in case of traumatic event or resective surgery). Immediate orbital floor repair was excluded.

All the patients attended Oral and Maxillofacial Unit at IRCCS Azienda Ospedaliero-Universitaria di Bologna from December 2009 to January 2024.

The patients were divided into three pathological groups: post-traumatic, post-oncological resection, and syndromic.

All the post-traumatic patients had undergone facial trauma in the previous months or years, determining an orbital floor fracture—“blow-out” type.

Patients affected by post-resective enophthalmos for oncologic included in this study had undergone the removal of important bony and muscular structures that are fundamental to guarantee the correct orbital protrusion, leading to worsening enophthalmos years after surgery.

Two patients included in the study are affected by Silent Sinus Syndrome (SSS), characterized by the congenital involution of the maxillary sinus, causing enophthalmos and/or hypoglobus.

Patients’ characteristics are detailed in Table 1.

For the selected cases, we have made an explicit request to the board of Medical Direction of our Hospital, illustrating the characteristics of each case and the reasons why our staff considered the patients worthy of being treated with a PSI: after receiving explicit authorization to insert them, we started the clinical and engineering workflow to produce the custom-made implants.

The reconstructive protocol involved 4 steps: mirroring of the healthy orbit on the affected side, virtual design of a patient-specific non-resorbable orbital floor mesh, CAM procedures for producing the customized implant, and surgical insertion of the device.

### 2.1. Virtual Planning and Computer-Aided Design

Planning began by acquiring a high-resolution CT scan of the patient’s craniofacial skeleton. Imaging was performed using a multidetector CT scanner (Lightspeed VCT LS, Advantage 64 slices, General Electric Medical System). Volumetric, DICOM-format data (0.625-mm slice thickness, 0.312 slice spacing, 08 gantry tilt, 512 × 512-pixel resolution) were processed using Proplan CMF™ and Mimics Innovation Suite 20.0 software (Materialise, Leuven, Belgium). After setting a suitable threshold value (Window Width = 4000 Hounsfield Units and Window Level = 400 Hounsfield Units), this software allows creation of three-dimensional virtual models of the maxillofacial skeleton.

**Figure 1:**
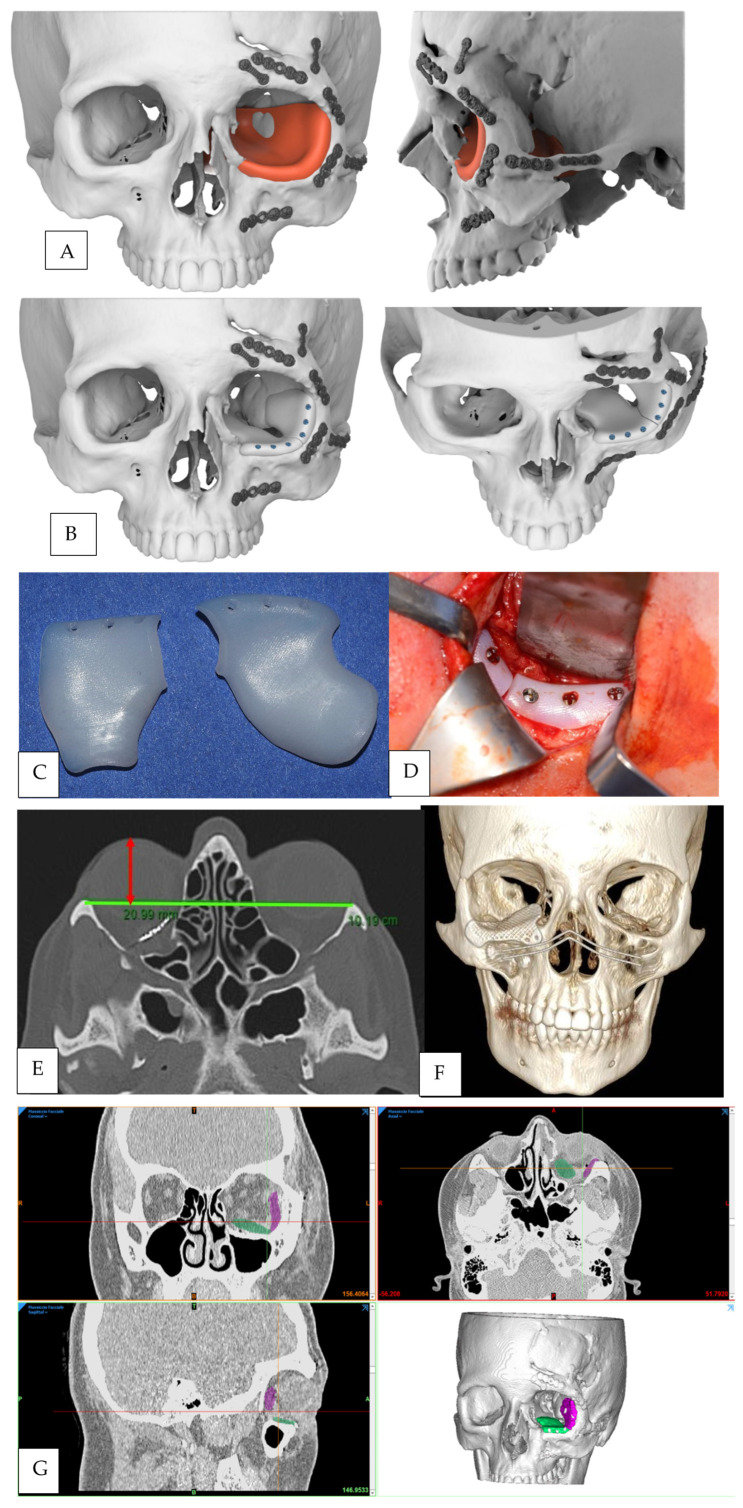
(**A**) Mirroring of the healthy bone surface on the affected side using 3D reconstruction of the CT scan. (**B**) CAD of the reconstructive left orbital floor implant. (**C**) High-density polyethylene implant in two pieces. (**D**) intraoperative image showing left orbital floor reconstruction via a transconjunctival approach through HDPE implant insertion. (**E**) Technique of proptosis measurement with CT as proposed by Ramli [18]. (**F**) Postoperative three-dimensional CT scan showing right orbital floor reconstruction using a customized titanium mesh. (**G**) 3D segmentation of the postoperative CT scan showing positioning of the HDPE implant with two different colors indicating each component of the prosthesis (green: medial component of the implant, purple: lateral component of the implant).

The CT scan was evaluated, and the normal uninjured side of the craniofacial skeleton was reflected onto the contralateral injured side by a mirroring technique using Geomagic Freeform 2016 (3D Systems, Rock Hill, SC, USA) (Figure 1A). A reconstructive orbital floor implant was then designed virtually on the mirrored orbital bone surface (Figure 1B).

### 2.2. Manufacturing of Customized Implants

Customized titanium meshes were surgically implanted in 11 cases.

Nonporous high-density polyethylene (HDPE) implants were used in 2 patients (Figure 1C).

The custom-made implants were generally as thick as the overlay obtained by the virtual mirroring of the healthy orbit upon the injured one, with an average thickness of 1.5 mm. This thickness also takes into account the presence of the inferior oblique muscle in the dissection, which leads to orbital floor exposure so that the implant does not interfere with the muscle.

Solid-to-layer files were used to manufacture the titanium mesh by direct metal laser-sintering (Sintac srl, Trento, Italy), which resolves the shaping and bending biases inherent in the indirect method (using an anatomical model) [19].

Computer Numerical Control (CNC) machining, a manufacturing process in which pre-programmed computer software dictates the movement of factory tools and machinery, was instead used for the manufacturing of the HDPE implants.

All PSI used were planned and manufactured according to Regulation (EU) 2017/745 of the European Parliament and the Council of 5 April 2017 on medical devices.

### 2.3. Surgical Procedure

Transconjunctival lower-eyelid approach, associated with lateral canthotomy and cantholysis to obtain a wider surgical field, was performed in 10 cases (Figure 1D); in just 1 case, isolated transconjunctival approach alone was executed. Weber-Ferguson incision was performed in 2 complex post-oncological reconstructive cases, of which one was associated with Lynch subciliary extension and the other one with Dieffenbach subciliary extension: this approach was chosen for this case because of the soft tissue fibrosis following the previous surgeries (expected from both clinical and radiological analysis), that could make a transconjunctival approach much difficult in terms of dissection of the tissues.

After dissection of the inferior orbital rim and opening of the periosteum, the patient-specific implant was fixed onto the intact facial buttress. More specifically, after periosteum incision, a subperiosteal dissection is carried. Even though using a transconjunctival approach usually means encountering and interfering in the lower oblique muscle, this one can be displaced and/or risen or maintained without damage to it, especially when there is no involvement of the medial orbital wall [20]. After exposure of the orbital floor defect with identification and preservation of the inferior oblique muscle and the inferior rectus muscle, the customized device was placed on the defect and further fixed with 1.5 mm diameter screws as needed. Inferior oblique muscle is eventually placed over the implant if it is encountered and risen.

A forced duction test was performed at the end of the surgical procedure to verify the absence of muscular entrapment and the correct ocular movement.

Surgical site was then closed by layered sutures according to the different approaches.

### 2.4. Outcomes Evaluation

Preoperative and postoperative globe protrusions were assessed as described by Ramli et al. [18]. All patients underwent CT scans of the orbit after surgery (from 24 to 72 h after surgery). Each patient had their head strapped in the supine position during the scan to ensure reliability of the measurements. Their positions were centered by aligning the midsagittal line perpendicular to the laser line. The coronal line was centered 1 cm above the external auditory meatus.

An interzygomatic line was first drawn on the axial-view image in which the lens was best seen. A perpendicular line was then drawn from the inner corneal surface to the interzygomatic line, bisecting the lens. The length of this perpendicular line was taken as the primary measurement (Figure 1E). As proposed by Ramli [18], Hertel exophthalmometer and CT measurements of globe position are similar and strongly correlated.

The globe protrusion measurements were made separately by two different clinicians, and a mean value between the two was considered.

We have decided to use the millimetric difference (Δ) of globe protrusion between the affected and the healthy side detected in CT scans according to Ramli’s method as a parameter of predictability and reproducibility of the whole CAD-CAM procedure.

Postoperative 3D CT scans were also evaluated to verify the correct positioning of the implant (Figure 1F).

For the two cases treated by positioning of HDPE implants, we performed a segmentation of postoperative CT scans in order to obtain information about the correct positioning of the implants, which cannot be easily detectable in a direct 3D reconstruction because of the radiolucent properties of HDPE (Figure 1G).

Aesthetical outcomes were assessed using 3dMD photogrammetry (3dMD Inc., Atlanta, GA, USA). Faces were captured using the 3dMD photogrammetric system, with patients keeping their eyes open.

The three-dimensional facial surface was calculated using 3dMD patient software (version 3.0). The Virtual Reality Modeling Language file with colored texture was used for the final virtual face display. Measurements of facial symmetry, globe protrusion, and dystopia were clinically analyzed.

The mean surgical procedural time was registered for each patient.

### 2.5. Control Group

A control group consisting of ten patients treated for secondary orbital floor reconstruction using a stock titanium mesh has been considered as the gold standard procedure to compare our experimental results.

Seven out of ten patients were treated for post-traumatic sequalae; three out of ten were post-oncologic cases.

All patients were treated by performing the same surgical procedures used in the test group. The titanium mesh was manually bent intra-operatively.

## 3. Results

The average age of patients undergoing surgery was 40.3 years old (range 18–86) in the test group and 40.5 (range 25–73) in the control group.

The mean follow-up duration was 30.69 months (range 3–120) and 25.30 (8–95) in the control group.

The mean surgical procedural time was 74 min (range 60–100) for the test group and 90 min (70–110) for the control group.

No acute or delayed implant-related complication, including wound site infection, dehiscence, hematoma, foreign body reaction, and diplopia, has been observed.

Edema of the surgical site, involving periorbital soft tissue, has been observed in every patient, lasting from 3 to 6 days after surgery, treated with the administration of topical steroid collyrium. No tarsorrhaphy was required after surgery, and the patients could open their eyelids immediately after awakening from general anesthesia.

No reoperation was necessary to reposition any displaced implant in the immediate or in the delayed postoperative time.

All of the patients had shown no functional or visual impairment after surgery.

Clinical assessments showed that the three-dimensional mirroring technique and PSI were effective in obtaining high precision in orbital floor restoration if compared with the preoperative situation.

Photogrammetry comparison between pre- and post-operative confirmed total or subtotal restoration of globe symmetry between the two sides and the eye protrusion.

For the test group, globe protrusion, assessed using the Ramli method, appeared to be improved in all cases treated (Table 1): we have calculated this parameter, obtaining an improvement of 3.04 mm (range 0.3–6 mm).

**Figure 2:**
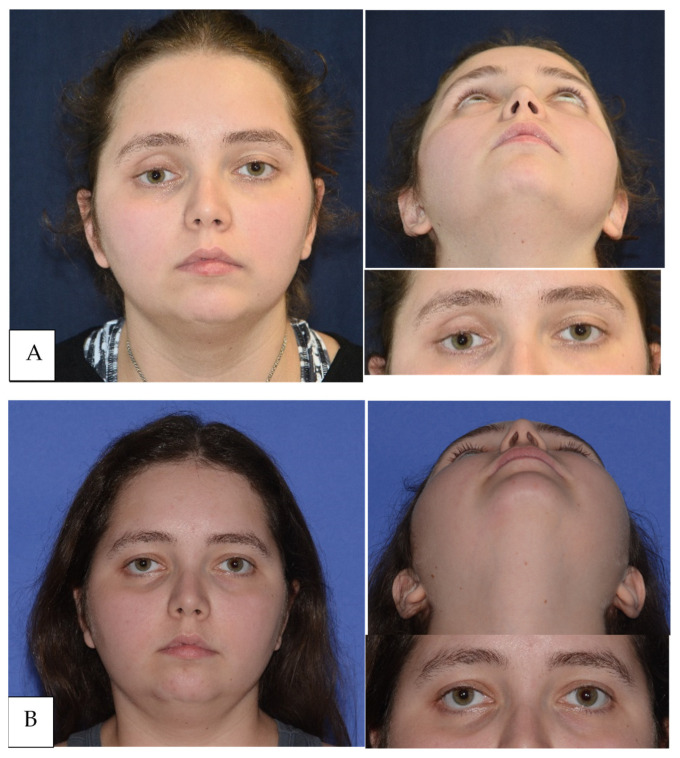
(**A**) Clinical assessment of right enophthalmos and dystopia in a frontal and inferior view. Clinical situation before orbital floor reconstruction. (**B**) Postoperative images showing clinical improvement of globe protrusion.

The Δ of postoperative globe protrusion between uninjured and treated eye was calculated: the mean Δ measured was 1.14 mm (range 0.27–3.69 mm), thus showing a mean difference between healthy and affected size < 2 mm in all cases, which is considered the minimum distance clinically detectable and evident by inspection [21] (Figure 2).

Further pictures to compare preoperative and postoperative clinical aspect of the patients treated with PSI can be found in the Supplementary Materials Figures S1 and S2.

For the test group, the mean Δ measured was 2.4 mm (range 0.42–3.9 mm).

No long-term complication has been registered, and no relapse of enophthalmos has been observed, except for only one oncological case (#3 from Table 1) due to the involution of soft tissue caused by the recurrence of the neoplasm in the following years.

## 4. Discussion

A large number of studies have searched for the optimal material for orbital reconstruction: it should have various characteristics, among which some of the most important are structural properties that guarantee long-term duration without deformation, high biocompatibility to avoid acute or late reject or infective complications, and a reasonable cost-effectiveness ratio.

Van Leeuwen et al. [22] have developed a mathematical model to preoperatively assess the suitability of implant materials according to defect size and help the surgeon properly choose the minimal thickness of reconstruction materials in order to avoid orbital volume increase and consequent enophthalmos.

A review made by Dubois et al. [23] satisfactorily shows the advantages and disadvantages of various materials employed in orbital reconstruction, emphasizing the properties of titanium implants in terms of stability, duration, and integration among orbital tissues: it can be safely used even for large defects of the orbit, maintaining a high level of biocompatibility with predictable results over the time.

Other studies state the reliable properties of polyethylene implants, showing satisfactory surgical outcomes and infection rates similar to autografts over the years [24].

Porous polyethylene has been used over the past decades as one of the main materials for craniomaxillofacial surgery procedures, thanks to its high biocompatibility, duration, and malleability: it is easy to mold but keeps its strength, so it offers the possibility to obtain a precise three-dimensional shape. Orbital floor reconstruction remains one of the most common applications of porous polyethylene shaped as thin (1.5 mm) or ultrathin (0.85 mm) sheets.

Materials used for orbital reconstruction can be categorized into resorbable and not resorbable; we only used non-resorbable materials in this study because of the long-term duration and predictability over time that they can give.

We have decided not to use autologous bone grafts because of the higher morbidity rates of the donor site [25] and the major rate of resorption over time that can lead to a relapse of enophthalmos. The morbidities generally connected to autologous bone graft harvesting are wound dehiscence or infections, prolonged acute pain, and loss of sensitivity in the area of the donor site, such as lateral femoral cutaneous nerve for the harvesting of anterior iliac crest [25].

A review made by Saluja et al. about orbital floor reconstruction using autologous bone graft [26] shows how one of the main concerns about the use of autologous bone graft is the likelihood of having a certain rate of resorption over time, which is quite unpredictable: it seems to be more frequent with the use of iliac crest because of the predominant cancellous bone composition of the bone harvested, while calvarium appears to be a safer and more predictable donor site, subject to fewer resorption phenomena, thanks to the diploic vascular system of this bone.

Most authors in the past decades have conversely stated the superiority of autologous bone grafts in terms of predictability and biocompatibility for orbital reconstruction. Kelly et al. have conducted a retrospective study on 50 patients to analyze which bone graft is the most suitable for this purpose, obtaining better results with the calvarian bone graft [27].

Another work from Bande et al. [28] proposes the use of the anterior wall of the maxillary sinus to reconstruct the orbital floor either in the early stage or the late reconstructive stage: this kind of graft appears to be feasible, easy to harvest, and offers at the same time a larger surgical field on the inferior side of the orbit from the intra-oral approach on the maxillary sinus, letting the surgeon have a larger manipulation of the site.

Porous high-density polyethylene has been widely used over the past decades as an alloplastic material for orbital floor reconstruction [29]: the main forms of this material are thin sheets (Medpor^®^, Stryker, Kalamazoo, MI, USA), associated or not with some titanium component to confer more strength and resistance. These sheets are available in a variety of sizes and in thicknesses ranging from 0.25 mm to 3.0 mm (even if they are generally used as 1.5 mm or 0.85 mm thickness sheets), and they can be molded and shaped by hot water. Their insertion and positioning can be obtained through a classical transconjunctival or subciliary incision. The displacement rate, which is a potential complication when using any implant, can be lowered by the use of fixation with a single thin screw on the inferior orbital rim. However, as Lin et al. state in their study [30], even though some authors advocate the advantages of screw fixation when using HDPE implants [31], clinical and scientific evidence demonstrates how a correct positioning of the implant, followed by closure of the periosteum at the inferior orbital rim, as well as orbital content pushing down, guarantee the stability of the sheets without the need of any further fixation. Moreover, fixation is not always allowed when comminution of the rim occurs and in the case of particularly thin orbital floor bones.

Medpor^®^ appears to be highly biocompatible because of the fibrovascularization that is created over time among its fibers, giving the implant a high duration and low rates of infection because of the consequent increased immune response mediators flow at the site.

Fibrovascularization can be demonstrated by contrast enhancement in the orbital implant site at CT or Magnetic Resonance Imaging (MRI) scans [32].

In this case series, we propose the use of nonporous high-density polyethylene implants obtained from a computer-assisted manufacturing process through a CNC technique: these implants were composed of two pieces to be assembled for orbital floor reconstruction. The composition in two different pieces was made in order to obtain an easier positioning during surgery: transconjunctival access to the orbital floor does not provide a wide overview of the surgical field, so it can be challenging to insert a single patient-specific implant with a huge extension all over the orbital floor.

These implants are characterized by a greater thickness than classical Medpor^®^ sheets (1.5–2 mm), giving the chance to obtain a slightly greater amount of globe protrusion, as well as more strength over time. Moreover, the greater thickness of these implants (compared to the titanium ones) may avoid the formation of a dead space resulting from the positioning of a layer above an altered or low-positioned orbital floor: this helps in preventing the formation of seromas or ematomas that could increase the risk of infections.

Despite a short-term follow-up, we have observed that HDPE implants are easy to manufacture, being a medium-cost material when compared to titanium implants.

They are robust and adaptable at the same time, giving us the chance to minimally reshape them intraoperatively if it is necessary, which is not possible when a titanium mesh is used.

They appear to be biocompatible: we have not observed any acute or late-onset rejection, infection, or exposure of the implants.

The main advantages derived from CAD-CAM technologies in orbital reconstruction are the sparing of operative time and the greater accuracy in positioning, thus leading to better functional and morphological outcomes; moreover, CAD-CAM procedures require accurate preoperative planning, determining a larger amount of time spent by clinicians studying and preparing for the surgery on the single clinical case.

However, after an initial learning curve, the procedural time is significantly reduced. A study by Zieliński et al. [33] showed shorter operative time when individual implants were used and less intraoperative bleeding time in patients in whom individual implants were used compared to the use of titanium mesh for orbital reconstruction.

In our clinical study, the mean surgical procedural time was lower for patients treated with PSI compared to patients treated using stock implants.

Functional and aesthetic results were significantly satisfactory; all patients had obtained a valid globe repositioning assessed using 3dMD photogrammetry.

We have decided to use Δ of postoperative globe protrusion between uninjured and treated eyes as a measure of predictability of the CAD-CAM method, as the minimum value of Δ represents the optimal clinical results of enophthalmos resolution. It is a reliability parameter since the mean value obtained was <2 mm, which is the minimal clinically relevant difference of protrusion between the two eyes.

Although the number of patients enrolled is quite small, this outcome appeared to have improved compared to the results obtained from our control group.

Moreover, our results confirm the reliability and efficacy of the titanium implants, which appear to be strong, biocompatible, and capable of osseointegration over the years: they also have a high rate of predictability thanks to the absence of resorption and remodeling rate.

This study comes after a preliminary one [17] realized in our center and published in 2016 that represented a first step in the use of a CAD-CAM technique for orbital floor reconstruction. It showed good results in terms of reliability and accuracy in orbital floor reconstruction using patient-specific implants.

In our center’s experience, over the past few years, we have studied and observed that CAD-CAM technologies guarantee a high standard of accuracy and result in a very high degree of reproducibility in various fields of Oral and Maxillo-Facial reconstructive surgery.

They have been used for the reconstruction of complex areas following mandibular resections [34,35].

Nowadays, with the spread of digital technologies applied in surgical planning, thanks to early training for Residents as well, it appears increasingly easy to manage and obtain tailored planning and treatment for the patients, and it should be considered as a valid option whenever a reconstructive procedure is required.

More specifically, in orbital reconstruction, it is essential to accurately reproduce the patient’s anatomy for its esthetical—and consequent social role: it thus becomes evident how a custom-made procedure is more indicated for secondary reconstruction, as it represents a routine procedure and does not have to meet urgent times.

Finally, costs could be considered as a limit of the procedure. However, although the price for PSI outdoes the total cost of freehand reconstructions when all aspects related to the quality of results were considered, including intraoperative time reduction and the lowering of morbidity leading to reduced hospitalization rate, CAD-CAM procedures appear to be economically viable [36].

## 5. Conclusions

In conclusion, the CAD-CAM technique described in this study appears to be an effective method for correcting complex orbital floor defects causing enophthalmos.

We have confirmed the feasibility of a method for the 3D preoperative planning of custom-made implants thanks to the mirroring technique, which is currently the gold standard for obtaining a hypothetical model of a patient’s healthy anatomy.

Computer-aided manufacturing offers the opportunity to obtain virtually planned implants in a highly precise and predictable way, maintaining affordable costs of production for specific clinical cases. Thanks to the improvement in production times, we can obtain precision and efficiency in reconstruction reasonably rapidly.

We have planned, developed, and inserted innovative HDPE implants in two patients, showing the feasibility of this method and the reliability of a material that had never been used for the purpose of orbital floor reconstruction. We certainly need to reach longer follow-up times to assess the clinical and radiological long-term efficacy of the use of these implants, even if the preliminary results are satisfactory and promising.

In general, the use of patient-specific orbital implants has demonstrated once again a high level of accuracy and predictability, showing a very small difference between postoperative uninjured and injured globe protrusion, resulting in being clinically undetectable for most patients.

We believe that late or secondary corrections of the orbital floor should be performed using CAD-CAM technology for several reasons. It gives a more accurate prediction thanks to computer-aided virtual planning, which allows the construction of customized implants. Moreover, the step-by-step manufacturing procedure of the reconstruction plate through direct metal laser-sintering and the CNC technique allows the surgeon to obtain better procedural control and reduce operative time.

We have observed no acute or delayed clinical complications: our case series shows the biocompatibility of both titanium and nonporous high-density polyethylene implants and their stability over time.

## Figures and Tables

**Table 1:** Patients’ main features and clinical outcomes evaluation.

Patient	Age at Surgery	Pathology	Time between Primary Event and Late Reconstructive Surgery	Δ between Preoperative and Postoperative Globe Protrusion, mm	Δ between Postoperative Globe Protrusion between Healthy and Treated Eye, mm	Follow-Up, mo
#1	34	Trauma	36 mo	3.8	1.93	48
#2	29	Trauma	14 yrs	4.8	0.27	36
#3	41	Neoplasm	27 mo	0.3	0.50	120
#4	20	Trauma	18 mo	2.7	0.48	39
#5	75	Neoplasm	15 mo	3.5	2.22	48
#6	48	Trauma	19 yrs	2.6	0.97	36
#7	43	Trauma	24 mo	2.3	1.22	12
#8	45	Trauma	8 mo	0.6	0.33	2
#9	26	SSS *	26 yrs	2.0	0.68	17
#10	32	Trauma	17 mo	2.8	0.67	3
#11	18	SSS *	18 yrs	5.0	3.69	23
#12	27	Trauma	24 mo	6.1	1.72	12
#13	86	Trauma	10 mo	4.9	1.14	3

* SSS—Silent Sinus Syndrome.

## Data Availability

All the data are available upon request, with permission for research and scientific purposes.

## Supplementary Materials

The following supporting information can be downloaded at: https://www.mdpi.com/article/10.3390/jpm14050459/s1, Figure S1: (A) a lady with left eyeball dystopia and enophthalmos due to a previous craniofacial oncological resection. She had both vertical diplopia and in primary position. (B) After orbital reconstruction, she obtained a good both resolution of eyeball dystopia and diplopia. Figure S2: (A) a lady affected by post-traumatic enophthalmos without diplopia. (B) She had a floor of the orbit reconstruction, obtaining a correct eyeball repositioning, maintaining the correct muscular function.
