# Supplementary material for: Delayed Orbital Floor Reconstruction Using Mirroring Technique and Patient-Specific Implants: Proof of Concept
Source: J Pers Med. 2024 Apr 26;14(5):459. doi: 10.3390/jpm14050459 (PMC11122088; doi:10.3390/jpm14050459)
Supplement: Supplementary file 1 [file jpm-14-00459-s001.zip › jpm-2969225-supplementary.pdf]

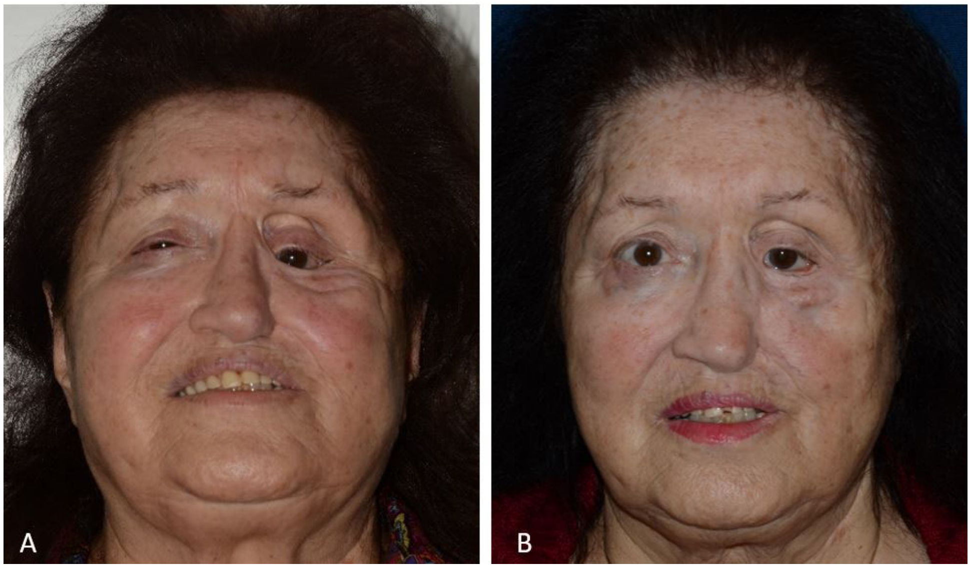

Figure 1: A) a lady with left eyeball dystopia and enophthalmos due to a previous craniofacial oncological resection. She had both vertical diplopia and in primary position. B) After orbital reconstruction, she obtained a good both resolution of eyeball dystopia and diplopia.

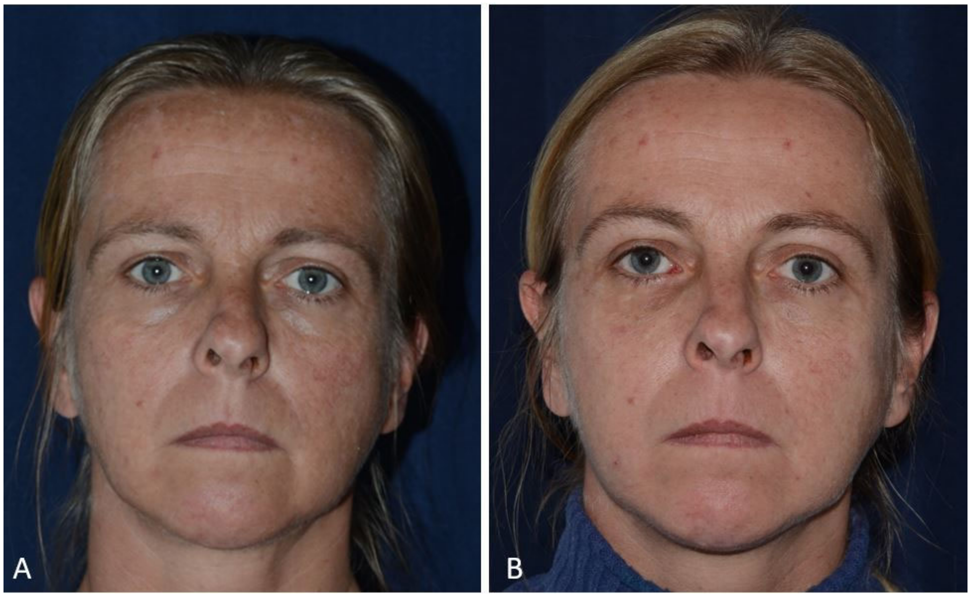

Figure ~~3~~2: A) a lady affected by post-traumatic enophthalmos without diplopia. B) She had a floor of the orbit reconstruction, obtaining a correct eyeball repositioning, maintaining the correct muscular function.

Commented [M1]: Figures should be in numberical order, we revised Figure 3 to Figure 2, please confirm.
